# Trends in atrial fibrillation and flutter mortality associated with disorders of thyroid gland in the United States from 1999 to 2020

**DOI:** 10.1002/joa3.70096

**Published:** 2025-05-22

**Authors:** Shahzaib Ahmed, Shoaib Ahmad, Hamza Ashraf, Eeman Ahmad, Umar Akram, Abbas H. Mallick, Irfan Ullah, Raheel Ahmed, Chadi Alraies, Gregg C. Fonarow

**Affiliations:** ^1^ Department of Medicine Fatima Memorial Hospital College of Medicine and Dentistry Lahore Pakistan; ^2^ St. Joseph Hospital and Medical Center Phoenix Arizona USA; ^3^ Department of Medicine Allama Iqbal Medical College Lahore Pakistan; ^4^ Department of Internal Medicine The Brooklyn Hospital Center Brooklyn New York USA; ^5^ Department of Internal Medicine Khyber Teaching Hospital Peshawar Pakistan; ^6^ National Heart and Lung Institute Imperial College London London UK; ^7^ Department of Cardiology Detroit Medical Center Detroit Michigan USA; ^8^ Division of Cardiology University of California Los Angeles California USA

**Keywords:** atrial fibrillation, atrial flutter, disorders of thyroid gland, mortality

## Abstract

**Background:**

Thyroid gland disorders are a known risk factor for atrial fibrillation and flutter (AF/AFL). Despite being a well‐established risk factor, most studies have primarily examined prevalence, comorbidities, and treatment patterns with little to no research on mortality trends for this association. Objective: We aimed to analyze the trends in AF/AFL‐related mortality in patients with thyroid gland disorders.

**Methods:**

Age‐adjusted mortality rates and crude rates per 100,000 population from 1999 to 2020 using the CDC WONDER database. Annual percent changes and their averages were calculated via Joinpoint regression. AF/AFL‐related mortality trends in patients with thyroid disorders were compared with those in the general population using pairwise comparison.

**Results:**

In the study period, a total of 7187 AF/AFL‐related deaths were observed in individuals diagnosed with thyroid gland disorders. The age‐adjusted mortality rates increased throughout the study period. The mortality rates in females remained consistently higher than those in males. Mortality rates did not vary substantially across regions (South: 0.09; Northeast: 0.09; Midwest: 0.10; West: 0.11). Furthermore, the annual percent change in females and South with thyroid disorders differed significantly from the general population. The states with the highest mortality rates were Oregon, Wyoming, and Nebraska. The mortality rates remained higher in nonmetropolitan regions (0.11) than in metropolitan regions (0.09).

**Conclusions:**

AF/AFL‐related mortality trends associated with thyroid disorders increased from 1999 to 2020. Policies that target vulnerable populations and regions may be beneficial in mitigating the increasing AF/AFL‐related mortality associated with disorders of the thyroid gland.

## Graphical abstract

Significant increase in mortality rates during the first decade (1999–2008), with an annual percent change of 7.80%, followed by a moderate increase from 2008 to 2020. Females had a higher mortality rate compared to males. Among racial groups, NH White individuals had the highest mortality rates.

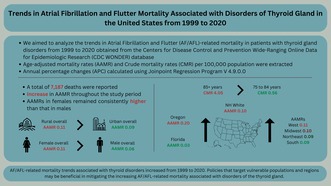

## 1 INTRODUCTION

Atrial fibrillation (AF) and atrial flutter (AFL) are common cardiac arrhythmias and pose significant mortality and morbidity risk. The burden of AF and AFL has been steadily increasing over the past few years and the prevalence of AF is estimated to have increased by threefold according to the Framingham Heart Study (FHS).^1^, ^2^ This increase in prevalence can be due to various reasons with the increase in AF risk factors like obesity, age, and hypertension thought to be major contributors.^3^

Thyroid hormones play a crucial role in regulating cardiac contractility, heart rate, and vascular resistance. Hyperthyroidism and hypothyroidism have both been linked with cardiac arrhythmias, and hyperthyroidism, in particular, is associated with an increased risk of developing AF and AFL. Recent studies have shown the role of hypothyroidism in AF‐related morbidity and mortality, where it has been documented to be associated with increased hospitalization rates for AF‐related complications and heart failure. In addition, elevated TSH levels and reduced T3 levels have been linked to higher risks of cardiovascular and all‐cause mortality.^4^

An analysis of the JoFib registry highlighted a 10.5% prevalence of thyroid disorders among atrial fibrillation (AF) patients, with hypothyroidism being the predominant type (90%) followed by hyperthyroidism (6.1%). Patients with thyroid dysfunction demonstrated higher rates of comorbidities such as pulmonary hypertension and chronic kidney disease.^5^

Despite the established association between thyroid dysfunction and AF/AFL, most studies have primarily focused on prevalence, comorbidities, and treatment patterns. However, there is little literature available regarding the relationship between thyroid disorders and AF/AFL‐related mortality. This study addresses these gaps by analyzing long‐term trends in AF/AFL‐related mortality associated with thyroid disorders, with a focus on regional, demographic, and gender‐based variations.

## 2 METHODS

### 2.1 Study design

We obtained death certificate data from the Centers for Disease Control and Prevention Wide‐Ranging Online Data for Epidemiologic Research (CDC WONDER) database to analyse AF/AFL‐related mortality in patients with thyroid disorders between 1999 and 2020.^6^ We used International Classification of Diseases, 10th Revision (ICD‐10) E00‐E07 (disorders of thyroid gland) and I48 (AF/AFL) for this analysis.^7^ This study included all deaths with thyroid disorders as a contributing cause of mortality and AF/AFL‐related disease as a primary or underlying cause of mortality. The underlying cause of death (i.e., AF/AFL) is defined as the disease that is the primary and direct cause leading up to death, whereas the contributing causes of death (i.e., thyroid disorders) are defined as any other conditions that the patients presented with that are associated with death. This study utilized anonymized, publicly available data, thus it did not require ethical approval from an Institutional Review Board (IRB). Our study complied with the Strengthening the Reporting of Observational Studies in Epidemiology (STROBE) guidelines.^8^

### 2.2 Data abstraction

Data collected included year, population, demographics, location of death, urban–rural classification, region, and states. Demographic variables included gender, age groups, and race/ethnicity. Locations of death encompassed medical facilities, homes, hospices, and nursing homes/long‐term care facilities. Race/ethnicity categories were non‐Hispanic (NH) White, NH Black or African American, Hispanic or Latino, NH American Indian or Alaskan Native, and NH Asian or Pacific Islander, as reported on death certificates. Regions were classified as Northeast, Midwest, South, and West per US Census Bureau definitions, and we used the National Center for Health Statistics Urban–Rural Classification Scheme (2013) to categorize areas as urban (large metropolitan areas with populations ≥1 million, medium/small metropolitan areas with populations between 50,000–999,999), or rural (population <50,000).^9^

### 2.3 Statistical analysis

To assess AF/AFL‐related mortality trends in patients with thyroid disorders, we calculated age‐adjusted mortality rates per 1,000,000 population, with 95% confidence intervals (CIs), standardized to the US population in the year 2000.^10^ Temporal trends were analyzed using the Joinpoint Regression Program (Joinpoint V 4.9.0.0, National Cancer Institute), which calculates the annual percent change in age‐adjusted mortality rates with 95% CIs.^11^ This method identifies significant changes by fitting log‐linear regression models and marking years of notable variation with joinpoints.^11^ Annual percent changes were considered increasing or decreasing if the slope for mortality change significantly differed from zero, assessed using a *t*‐test. Statistical significance was set at a two‐tailed *p* < 0.05. A pairwise comparison was performed to evaluate the significance of the differences in the percentage change between the two cohorts (thyroid disorders‐contributing AF/AFL‐related mortality and all AF/AFL‐related mortality) stratified by the various factors (such as gender, race, and age categories).^12^ This study was exempt from institutional review board approval as it relied on de‐identified public data from the government. Any data suppressed in the CDC‐WONDER database for confidentiality reasons were excluded from the analysis.

## 3 RESULTS

**FIGURE 1:**
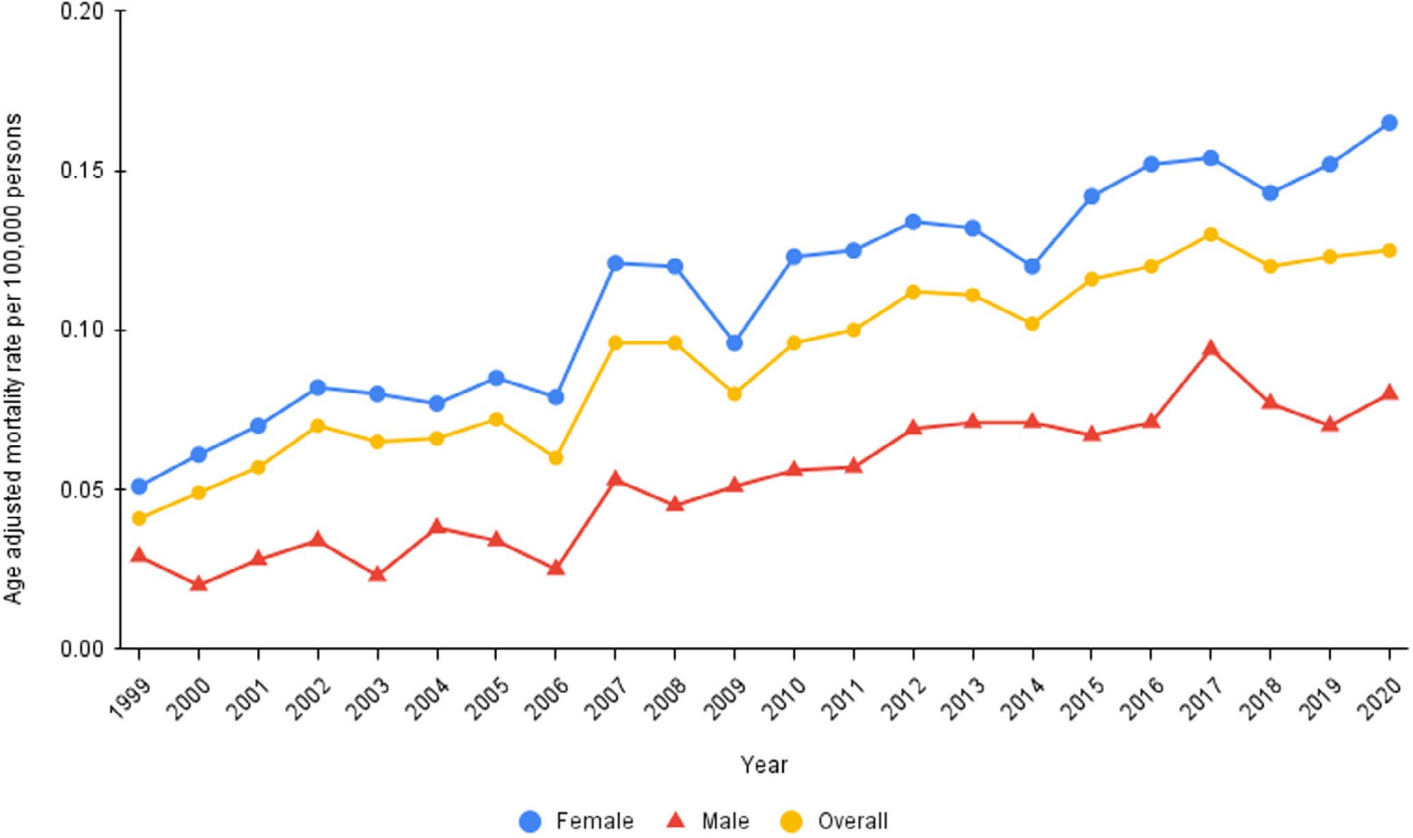
Figure shows increasing overall and gender‐stratified trends in atrial fibrillation and flutter‐related mortality in patients with thyroid disorders, age‐adjusted mortality rates per 100,000 individuals in the United States from 1999 to 2020.

During the study period, a total of 7187 AF/AFL‐related deaths were observed in individuals diagnosed with thyroid gland disorders, with an average age‐adjusted mortality rate of 0.097 (95% CI: 0.095–0.099) per 100,000 (Table 1). The cohort experienced a substantial increase in mortality from 1999 to 2008 (Annual percent change: 7.80*; 95% CI: 4.25–11.46), followed by a moderate increase from 2008 to 2020 (Annual percent change: 2.93*; 95% CI: 1.41–4.47) (Figure 1, Table 2). The AF/AFL‐related mortality in people with thyroid disease did not differ significantly when compared with AF/AFL alone (*p* = 0.29, Average annual percent change: 4.99*; 95% CI: 3.38–6.62 and 3.62*; 95% CI: 3.09–4.15, respectively) (Table 3). AF/AFL‐related mortality in patients with thyroid disorders was only 1.9% of that in the overall population (Rate Ratio: 0.019; 95% CI: 0.019–0.019).

**TABLE 1:** Demographic characteristics of deaths due to atrial fibrillation and flutter‐related mortality in patients with thyroid disorders in the United States from 1999 to 2020.

Variable	Deaths	Population	Age‐adjusted mortality rate (95% CI)
Overall	7187	6,746,356,647	0.097 (0.095–0.099)
Gender			
Female	5587	3,429,003,804	0.114 (0.111–0.117)
Male	1600	3,317,352,843	0.057 (0.054–0.06)
Age groups^a^			
75–84 years	1675	298,504,433	0.561 (0.534–0.588)
85+ years	4844	119,513,891	4.053 (3.939–4.167)
Race/ethnicity			
NH White	6581	4,394,181,258	0.103 (0.101–0.106)
Census region			
Northeast	1397	1,212,994,922	0.092 (0.087–0.097)
Midwest	1803	1,466,121,214	0.103 (0.098–0.108)
South	2282	2,497,818,081	0.085 (0.081–0.088)
West	1705	1,569,422,430	0.106 (0.101–0.112)
Urbanization			
Urban	5650	5,739,475,649	0.088 (0.086–0.09)
Rural	1537	1,006,871,652	0.111 (0.105–0.117)
Place of death^b^			
Medical facility	2193	NA	NA
Decedent's home	1795	NA	NA
Hospice facility	196	NA	NA
Nursing home/long‐term care	2663	NA	NA

Abbreviation: CI, confidence interval.

^a^ Crude mortality rate is used for analysis instead of age‐adjusted mortality rates for age groups.

^b^ Age‐adjusted mortality rates and population are not applicable for place of death.

**TABLE 2:** Annual percent changes and average annual percent change in atrial fibrillation and flutter‐related mortality in patients with thyroid disorders in the United States from 1999 to 2020.

Variable	Trend segment	Lower endpoint	Upper endpoint	Annual percent change (95% CI)	Average annual percent change (95% CI)	*p*‐value
Entire cohort	1	1999	2008	7.80^a^ (4.25–11.46)	4.99^a^ (3.38–6.62)	<0.000001
	2	2008	2020	2.93^a^ (1.41–4.47)		
Gender						
Female	1	1999	2008	7.90^a^ (4.58–11.33)	5.13^a^ (3.60–6.68)	<0.000001
	2	2008	2020	3.10^a^ (1.62–4.59)		
Male	1	1999	2017	7.14^a^ (5.50–8.80)	5.34^a^ (2.99–7.76)	0.000006
	2	2017	2020	‐4.81 (−17.51 to 9.86)		
Age groups						
75–84 years	1	1999	2020	4.18^a^ (3.19–5.18)	4.18^a^ (3.19–5.18)	<0.000001
85+ years	1	1999	2008	8.38^a^ (4.82–12.05)	5.25^a^ (3.66–6.87)	<0.000001
	2	2008	2020	2.97^a^ (1.53–4.43)		
Census region						
Northeast	1	1999	2020	4.97^a^ (3.90–6.05)	4.97^a^ (3.90–6.05)	<0.000001
Midwest	1	1999	2020	3.93^a^ (2.81–5.06)	3.93^a^ (2.81–5.06)	<0.000001
South	1	1999	2008	10.78^a^ (6.04–15.74)	5.88^a^ (3.79–8.02)	<0.000001
	2	2008	2020	2.35^a^ (0.48–4.25)		
West	1	1999	2016	6.10^a^ (4.79–7.44)	4.47^a^ (2.85–6.11)	<0.000001
	2	2016	2020	‐2.21 (−8.85 to 4.91)		
Urbanization						
Urban	1	1999	2007	8.65^a^ (4.73–12.72)	4.91^a^ (3.39–6.46)	<0.000001
	2	2007	2020	2.67^a^ (1.48–3.88)		
Rural	1	1999	2012	7.75^a^ (4.97–10.60)	5.70^a^ (3.66–7.78)	<0.000001
	2	2012	2020	2.45 (−1.10 to 6.12)		

Abbreviation: CI, confidence interval.

^a^ Indicates that the annual percent change or average annual percent change is significantly different from zero at the *α* = 0.05 level.

**TABLE 3:** Pairwise comparison between atrial fibrillation and flutter‐related mortality in patients with thyroid disorders and overall atrial fibrillation and flutter‐related mortality rates in the United States from 1999 to 2020.

Variable	Atrial fibrillation/flutter‐related mortality rates in patients with thyroid disorders			Overall atrial fibrillation/flutter‐related mortality rates			*p*‐value for average annual percent change comparison
	Age‐adjusted mortality rate (95% CI)	Average annual percent change (95% CI)	*p*‐value	Age‐adjusted mortality rate (95% CI)	Average annual percent change (95% CI)	*p*‐value	
Overall	0.097 (0.095–0.099)	4.99^a^ (3.38–6.62)	<0.000001	5.09 (5.07–5.10)	3.62^a^ (3.09–4.15)	<0.000001	0.294889
Gender							
Male	0.057 (0.054–0.06)	5.34^a^ (2.99–7.76)	<0.000001	5.01 (4.98–5.03)	4.06^a^ (3.51–4.62)	<0.000001	0.114667
Female	0.114 (0.111–0.117)	5.13^a^ (3.60–6.68)	<0.000001	6.11 (6.02–6.21)	3.30^a^ (2.70–3.90)	<0.000001	0.268444
Age groups^a^							
75–84 years	0.561 (0.534–0.588)	4.18^a^ (3.19–5.18)	<0.000001	35.25 (35.04–35.46)	3.08^a^ (2.47–3.70)	<0.000001	0.481778
85+ years	4.053 (3.939–4.167)	5.25^a^ (3.66–6.87)	<0.000001	182.511 (181.75–183.28)	3.59^a^ (3.03–4.14)	<0.000001	0.261111
Census region							
Northeast	0.092 (0.087–0.097)	4.97^a^ (3.90–6.05)	<0.000001	4.88 (4.84–4.91)	2.91^a^ (2.31–3.52)	<0.000001	0.083111
Midwest	0.103 (0.098–0.108)	3.93^a^ (2.81–5.06)	<0.000001	5.37 (5.34–5.41)	4.08^a^ (3.44–4.72)	<0.000001	0.594667
South	0.085 (0.081–0.088)	5.88^a^ (3.79–8.02)	<0.000001	4.95 (4.92–4.98)	3.34^a^ (2.69–3.99)	<0.000001	0.042889^a^
West	0.106 (0.101–0.112)	4.47^a^ (2.85–6.11)	<0.000001	5.13 (5.09–5.16)	4.01^a^ (3.21–4.82)	<0.000001	0.904889
Urbanization							
Urban	0.088 (0.086–0.09)	4.91^a^ (3.39–6.46)	<0.000001	4.95 (4.93–4.96)	3.47^a^ (2.93–4.02)	<0.000001	0.344889
Rural	0.111 (0.105–0.117)	5.70^a^ (3.66–7.78)	<0.000001	5.69 (5.65–5.73)	4.28^a^ (3.59–4.98)	<0.000001	0.162889

^a^ Crude mortality rates are reported instead of age‐adjusted mortality rates for age groups.

### 3.1 Trends by Gender

Females with thyroid disorders had a higher AF/AFL‐related mortality rate compared to males (Age‐adjusted mortality rate: 0.11; 95% CI: 0.11–0.18 and age‐adjusted mortality rate: 0.06; 95% CI: 0.05–0.06, respectively) (Table 1). Mortality rates in males experienced a substantial increase from 1999 to 2017 (Annual percent change: 7.14*, 95% CI: 5.5–8.8). However, the trend reversed from 2017 to 2020 with a nonsignificant decrease (Annual percent change: −4.81, 95% CI: −17.51 to 9.86). From 1999 to 2008, female mortality rates increased significantly (Annual percent change: 7.9*; 95% CI: 4.58–11.33). This increase continued, although at a slower pace, from 2008 to 2020 (Annual percent change: 3.1; 95% CI: 1.62–4.59) (Figure 1, Table 2). The AF/AFL‐related mortality in people with thyroid disease did not differ significantly from that of AF/AFL alone in both males (*p* = 0.12) and females (*p* = 0.27) (Table 3).

### 3.2 Trends by race

When stratified by racial groups, NH Whites had the highest mortality rates (age‐adjusted mortality rate: 0.10, 95% CI: 0.10–0.11), followed by NH Black or African American individuals (age‐adjusted mortality rate: 0.04, 95% CI: 0.04–0.05). The lowest mortality rate was observed in NH Asians or Pacific Islanders (Age‐adjusted mortality rate: 0.03, 95% CI: 0.03–0.04) (Table 1). Trend analysis was not possible due to the data suppression policy of CDC WONDER.

### 3.3 Trends by census region

When stratified by census region, the West region had the highest mortality rate (age‐adjusted mortality rate: 0.11; 95% CI: 0.10–0.11), followed closely by the Midwest (age‐adjusted mortality rate: 0.10; 95% CI: 0.10–0.11). The Northeast had a slightly lower mortality rate (age‐adjusted mortality rate: 0.092; 95% CI: 0.087–0.097), while the South recorded the lowest rate (age‐adjusted mortality rate: 0.085; 95% CI: 0.081–0.088) (Table 1, Figure S1). In the Northeast and Midwest, mortality rates increased significantly from 1999 to 2020 (Annual percent change: 4.97*; 95% CI: 3.58–6.93 and 3.93*; 95% CI: 2.89–5.33, respectively). The South experienced a substantial increase from 1999 to 2008 (annual percent change: 10.78*; 95% CI: 7.21–23.72), followed by a nonsignificant increase from 2008 to 2020 (annual percent change: 2.35; 95% CI: −0.61 to 4.06). In the West, rates increased significantly from 1999 to 2016 (annual percent change: 6.10*; 95% CI: 5.17–11.67), but then decreased from 2016 to 2020 (annual percent change: −2.21, 95% CI: −12.45 to 3.92) (Figure S2, Table 2). The AF/AFL‐related mortality in people with thyroid disease did not differ significantly from that of AF/AFL alone in the Midwest, West, and Northeast (*p* = 0.59, 0.90 and 0.08 respectively). However, the rates differed significantly in the South (*p* = 0.04; Average annual percent change: 5.89; 95% CI: 3.79–8.0 in for AF/AFL‐related mortality in thyroid disease, compared to average annual percent change: 3.34*; 95% CI: 2.69–3.99 for AF/AFL mortality alone) (Table 3).

### 3.4 Trends by urbanization

**FIGURE 2:**
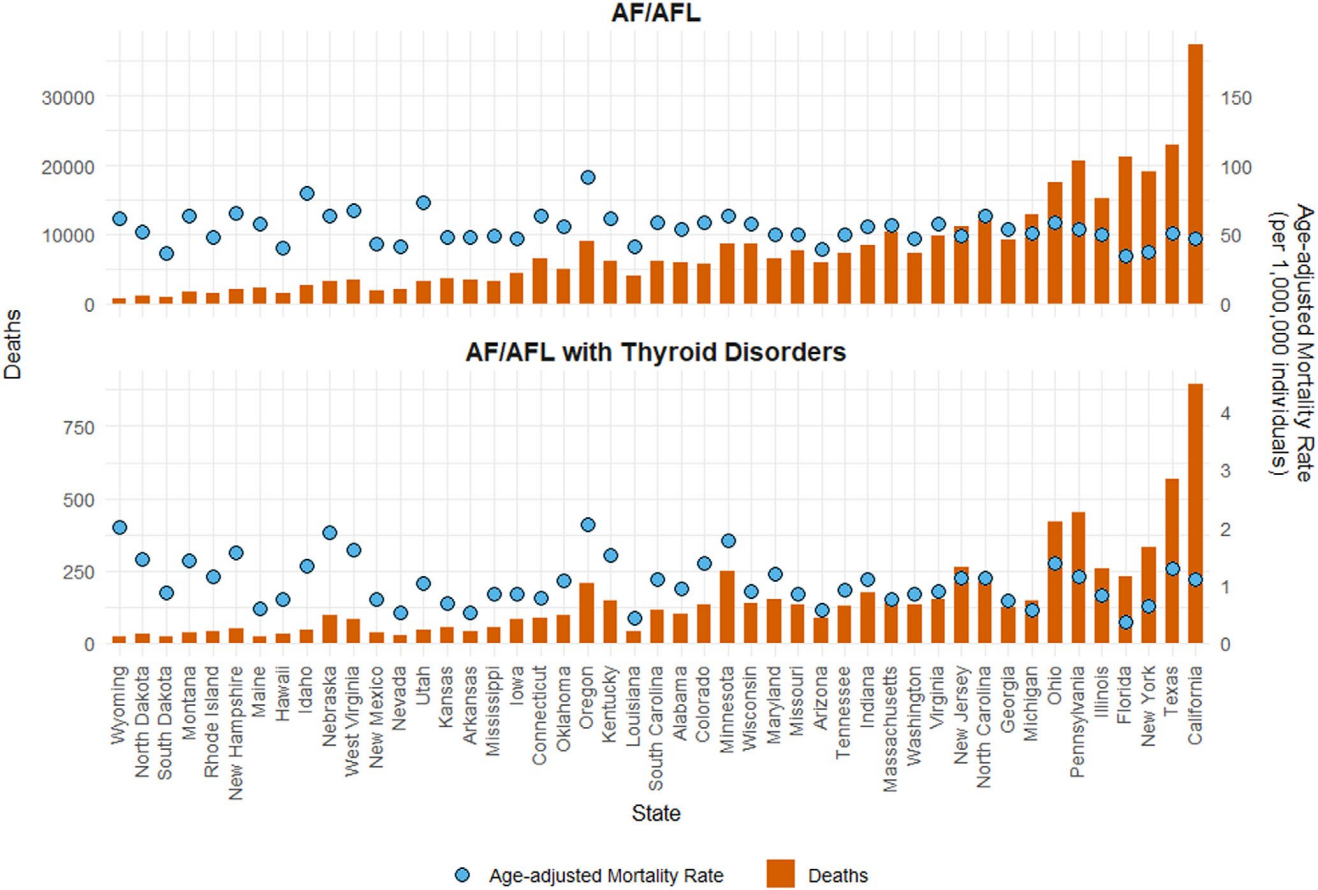
Figure shows state‐wise deaths and age‐adjusted mortality rates (per 100,000 population), arranged by population density, due to atrial fibrillation and flutter (AF/AFL) in patients with thyroid disorders in the United States from 1999 to 2020. AF/AFL: Atrial fibrillation and flutter.

Rural regions had a higher age‐adjusted mortality rate (0.11, 95% CI: 0.11–0.12) compared to urban regions (age‐adjusted mortality rate: 0.09, 95% CI: 0.086–0.090) (Figure S3, Table 1, Figure 2). Urban areas experienced a significant increase in mortality rates from 1999 to 2007 (annual percent change: 8.65*, 95% CI: 5.34–24.91), followed by another increase from 2007 to 2020 (annual percent change: 2.67, 95% CI: −0.67 to 3.93). Similarly, rural areas mortality rates also increased significantly from 1999 to 2012 (annual percent change: 7.75*, 95% CI: 5.55–46.90), followed by another increase from 2012 to 2020 (annual percent change: 2.45, 95% CI: −10.98 to 5.92) (Table 2). The average annual percent change of AF/AFL‐related mortality in people with thyroid disease did not differ significantly from that of AF/AFL alone in both urban and rural regions (*p* = 0.34 and 0.16, respectively) (Table 3).

### 3.5 Trends by age group

Age group 85 and above had a higher age‐adjusted mortality rate (4.05; 95% CI: 3.94–4.17) than the 75–84 age group (age‐adjusted mortality rate: 0.56; 95% CI: 0.53–0.59) (Table 1). Mortality rates for the 75–84 age group increased significantly from 1999 to 2020 (annual percent change: 4.18*, 95% CI: 3.29–5.31). For the 85 and above age group, there was a significant increase from 1999 to 2008 (annual percent change: 8.38*, 95% CI: 5.68–22.83), followed by another increase from 2008 to 2020 (annual percent change: 2.97, 95% CI: −0.13 to 4.27) (Figure S4, Table 2). The average annual percent change of AF/AFL‐related mortality in people with thyroid disease did not differ significantly from that of AF/AFL alone in the 75–84 and ≥85 age group (*p* = 0.48 and 0.26, respectively) (Table 3).

### 3.6 Trends by states

Differences in age‐adjusted mortality rates were prominent across different states. The states with the highest age‐adjusted mortality rate—Oregon (age‐adjusted mortality rate 0.202; 95% CI = 0.174–0.23), Wyoming (age‐adjusted mortality rate 0.2; 95% CI = 0.128–0.297), and Nebraska (age‐adjusted mortality rate 0.184; 95% CI = 0.149–0.226)—exhibited rates that were several times higher than those of states on the lower end of the spectrum. States with the lowest age‐adjusted mortality rates included Nevada (age‐adjusted mortality rate: 0.047; 95% CI = 0.03–0.072), Louisiana (age‐adjusted mortality rate: 0.038; 95% CI = 0.027–0.053), and Florida (age‐adjusted mortality rate: 0.032; 95% CI = 0.028–0.037) (Figure 2, Table S1).

## 4 DISCUSSION

Our study highlights the AF/AFL‐related mortality trends in individuals with thyroid disorders from 1999 to 2020. A total of 7187 deaths were reported, with an overall age‐adjusted mortality rate of 0.097 per 100,000. There was a significant increase in mortality rates during the first decade (1999–2008), with an annual percent change of 7.80% followed by a moderate increase from 2008 to 2020 (annual percent change: 2.93%). Females had a higher mortality rate (age‐adjusted mortality rate: 0.11) compared to males (age‐adjusted mortality rate: 0.06) and among racial groups, NH Whites individuals had the highest age‐adjusted mortality rate (0.10). The South region showed significantly higher AF/AFL‐related mortality in individuals with thyroid disorders compared to AF/AFL alone; however, there was no overall difference between the two. The age‐adjusted mortality rate remained higher in nonmetropolitan regions (0.11) as compared to metropolitan regions (0.09) throughout the study period.

There is conflicting evidence regarding the role of gender in the prevalence of AF/AFL in thyroid disease patients. In previous studies, men with hyperthyroidism have been documented to be at a greater risk of developing AF/AFL as compared to women, which is also considered true for individuals without thyroid diseases.^2^, ^13^ In the MISOAC‐AF study; however, most of the male patients with AF were euthyroid while more females with AF had hypothyroidism, and the difference was significant.^4^ In our study, females had a higher age‐adjusted mortality rate as compared to males. This is consistent with previous studies on AF/AFL mortality analysis which had no specific focus on individuals with thyroid disease.^14^, ^15^ Our findings also add more substance to an already published meta‐analysis on 4,371,714 patients where female patients as compared to men, despite having a lower AF prevalence, were found to have worse clinical outcomes with a higher risk of stroke, heart failure, cardiac events, and cardiovascular mortality.^16^

According to stratification by race, NH Whites had the highest mortality rate in our cohort, which is consistent with another broader study published on the general trends of AF/AFL mortality without a specific focus on individuals with thyroid diseases.^14^ Similar findings were present in another cohort study on 5201 patients, where blacks had a slightly lower risk of AF.^17^ However, the literature depicts well‐documented racial inequities in AF treatment, with minority populations often facing under‐detection, reduced access to advanced therapies, and underrepresentation in clinical trials.^18^ This disparity in mortality rates despite racial inequities can possibly be attributed to an increased prevalence of disease in the NH Whites. This theory can be supported by a previous study from the National Health and Nutrition Examination Survey (NHANES) database from January 1, 1999 to December 31, 2018 on 57,460 individuals that showed the highest prevalence of thyroid disorders in females, NH Whites, and individuals greater than 60 years.^19^ Hence, the higher age‐adjusted mortality rate in NH Whites in our study is likely multifactorial and may reflect an increased prevalence of both thyroid disorders and AF/AFL, better detection, and an older demographic profile in NH Whites.

Geographical stratification in our cohort revealed a higher age‐adjusted mortality rate in rural areas as compared to the urban regions. Certain Western and Midwestern states, including Oregon, Wyoming, and Nebraska, experienced disproportionately higher mortality rates that can possibly be attributed to an aging population and increased prevalence of various risk factors. The AF/AFL‐related mortality in people with thyroid disease did not differ significantly from that of AF/AFL alone in the Midwest, West, and Northeast. However, the rates differed significantly in the South. This can also likely be driven by differences in healthcare access, demographic profiles, and risk factor prevalence.

The mortality rates have been on a constant rise in each decade for AF/AFL in thyroid disease patients and our cohort experienced a substantial increase in mortality from 1999 to 2008, followed by a moderate increase from 2008 to 2020. This increase in mortality can be attributed to the increasing prevalence of thyroid diseases,^19^ the increasing prevalence of AF/AFL,^20^ and an aging population with an increased life expectancy.^1^, ^2^

The continual rise in mortality rates for individuals with thyroid disease having AF/AFL despite advanced treatments signals a growing concern. Future research should explore how risk factors impact progression, especially in aging populations. Improving access to therapies like endovascular ablation is critical, especially for the aging population. Moreover, addressing health care access disparities, particularly in underserved regions, is essential to reversing the upward trend in mortality.

This study utilizes a large dataset spanning two decades which provides a robust overview of mortality trends for AF/AFL in individuals with thyroid disease. However, certain limitations must be noted. Unreported cases and regional variations in healthcare access and quality may affect the generalizability of the findings. The study's lack of comprehensive data on possible confounders restricts the investigation of complicated variable associations. Moreover, certain conditions, especially those underdiagnosed or misclassified, may be underreported, leading to potential bias in trend analysis. Lastly, the retrospective design restricts the ability to predict future mortality trends that might be mitigated by addressing current disparities.

## 5 CONCLUSION

In conclusion, this study highlights significant differences in AF/AFL‐related mortality among individuals with thyroid disorders and emphasizes the compounded impact of gender, regional, and demographic factors. NH Whites and females exhibited the highest mortality rates, which likely reflect their higher disease prevalence, older demographic profile, and better detection rates. Some states showed significant differences in mortality trends, likely driven by differences in healthcare access and risk factor prevalence.

## AUTHOR CONTRIBUTIONS

**Shahzaib Ahmed:** conceptualization, project administration, formal analysis, writing—original draft, writing—review and editing; **Shoaib Ahmad:** conceptualization, writing—original draft, writing—review and editing; **Hamza Ashraf:** conceptualization, writing—original draft, writing—review and editing; **Eeman Ahmad:** conceptualization, writing—original draft, writing—review and editing; **Umar Akram:** conceptualization, writing—original draft, writing—review and editing; **Abbas H. Mallick:** conceptualization, writing—original draft, writing—review and editing; **Irfan Ullah**: conceptualization, writing—review and editing; **Raheel Ahmed:** conceptualization, writing—review and editing; **Chadi Alraies:** conceptualization, supervision, writing—review and editing; **Gregg C. Fonarow:** conceptualization, supervision, writing—review and editing.

## FUNDING INFORMATION

No funding was received for conducting this research.

## CONFLICT OF INTEREST STATEMENT

Dr. Fonarow reports consulting for Abbott, Amgen, AstraZeneca, Bayer, Boehringer Ingelheim, Cytokinetics, Eli Lilly, Johnson & Johnson, Medtronic, Merck, Novartis, and Pfizer. None of the other authors have any interests to declare.

## Supporting information


**Table S1.** State‐wise age‐adjusted mortality rates per 100,000 individuals and 95% confidence intervals (95% CIs) in atrial fibrillation and flutter‐related mortality in patients with thyroid disorders in the United States from 1999 to 2020.
**Figure S1.** Figure shows map of census region‐wise atrial fibrillation and flutter‐related mortality in patients with thyroid disorders in the United States from 1999 to 2020.
**Figure S2.** Figure shows age‐adjusted mortality rates per 100,000 individuals trends in atrial fibrillation and flutter‐related mortality in patients with thyroid disorders stratified by census region in the United States from 1999 to 2020.
**Figure S3.** Figure shows age‐adjusted mortality rates per 100,000 individuals trends in atrial fibrillation and flutter‐related mortality in patients with thyroid disorders stratified by urbanization in the United States from 1999 to 2020.
**Figure S4.** Figure shows age‐adjusted mortality rates per 100,000 individuals trends in atrial fibrillation and flutter‐related mortality in patients with thyroid disorders stratified by 10‐year age groups in the United States from 1999 to 2020.

## Data Availability

All data generated or analyzed during this study are included in this published article and its supplementary information files and are freely available on the CDC WONDER database.
