# Supplementary material for: Trends in atrial fibrillation and flutter mortality associated with disorders of thyroid gland in the United States from 1999 to 2020
Source: J Arrhythm. 2025 May 22;41(3):e70096. doi: 10.1002/joa3.70096 (PMC12096013; doi:10.1002/joa3.70096)

**Supplementary Material**

**Trends in Atrial Fibrillation and Flutter Mortality Associated with Disorders of Thyroid Gland in the United States from 1999 to 2020**

This supplemental material is provided by the authors for a better understanding of their work

**Supplementary Table 1:** State-wise Age-adjusted mortality rates per 100,000 individuals and 95% Confidence Intervals (95% CIs) in atrial fibrillation and flutter-related mortality in patients with thyroid disorders in the United States from 1999 to 2020

| State | Deaths | Age-Adjusted Mortality rates | Lower 95% Confidence Interval | Upper 95% Confidence Interval |
| --- | --- | --- | --- | --- |
| Alabama | 101 | 0.094 | 0.075 | 0.112 |
| Arizona | 86 | 0.06 | 0.048 | 0.075 |
| Arkansas | 39 | 0.054 | 0.038 | 0.075 |
| California | 897 | 0.109 | 0.102 | 0.117 |
| Colorado | 133 | 0.131 | 0.108 | 0.154 |
| Connecticut | 85 | 0.077 | 0.061 | 0.096 |
| Delaware | 14 | Unreliable | 0.032 | 0.104 |
| Florida | 231 | 0.032 | 0.028 | 0.037 |
| Georgia | 123 | 0.07 | 0.057 | 0.082 |
| Hawaii | 30 | 0.073 | 0.049 | 0.105 |
| Idaho | 46 | 0.128 | 0.094 | 0.172 |
| Illinois | 257 | 0.079 | 0.069 | 0.089 |
| Indiana | 173 | 0.108 | 0.091 | 0.124 |
| Iowa | 83 | 0.084 | 0.066 | 0.104 |
| Kansas | 53 | 0.069 | 0.051 | 0.091 |
| Kentucky | 148 | 0.145 | 0.121 | 0.169 |
| Louisiana | 41 | 0.038 | 0.027 | 0.053 |
| Maine | 23 | 0.057 | 0.036 | 0.086 |
| Maryland | 153 | 0.114 | 0.096 | 0.133 |
| Massachusetts | 139 | 0.07 | 0.058 | 0.083 |
| Michigan | 148 | 0.051 | 0.042 | 0.06 |
| Minnesota | 247 | 0.173 | 0.151 | 0.194 |
| Mississippi | 55 | 0.077 | 0.058 | 0.102 |
| Missouri | 133 | 0.085 | 0.07 | 0.099 |
| Montana | 38 | 0.137 | 0.096 | 0.188 |
| Nebraska | 96 | 0.184 | 0.149 | 0.226 |
| Nevada | 25 | 0.047 | 0.03 | 0.072 |
| New Hampshire | 52 | 0.146 | 0.108 | 0.193 |
| New Jersey | 262 | 0.109 | 0.096 | 0.123 |
| New Mexico | 34 | 0.077 | 0.053 | 0.108 |
| New York | 331 | 0.066 | 0.059 | 0.074 |
| North Carolina | 234 | 0.111 | 0.096 | 0.125 |
| North Dakota | 32 | 0.148 | 0.101 | 0.21 |
| Ohio | 419 | 0.129 | 0.117 | 0.142 |
| Oklahoma | 97 | 0.107 | 0.087 | 0.131 |
| Oregon | 207 | 0.202 | 0.174 | 0.23 |
| Pennsylvania | 453 | 0.112 | 0.102 | 0.123 |
| Rhode Island | 39 | 0.116 | 0.082 | 0.16 |
| South Carolina | 114 | 0.111 | 0.09 | 0.132 |
| South Dakota | 23 | 0.087 | 0.055 | 0.13 |
| Tennessee | 128 | 0.085 | 0.07 | 0.1 |
| Texas | 566 | 0.125 | 0.114 | 0.135 |
| Utah | 44 | 0.1 | 0.073 | 0.135 |
| Vermont | 13 | Unreliable | 0.043 | 0.138 |
| Virginia | 152 | 0.091 | 0.076 | 0.106 |
| Washington | 133 | 0.08 | 0.066 | 0.094 |
| West Virginia | 82 | 0.162 | 0.129 | 0.202 |
| Wisconsin | 139 | 0.084 | 0.07 | 0.099 |
| Wyoming | 24 | 0.2 | 0.128 | 0.297 |

**Supplementary Figure 1:** Figure shows map of census region-wise atrial fibrillation and flutter-related mortality in patients with thyroid disorders in the United States from 1999 to 2020
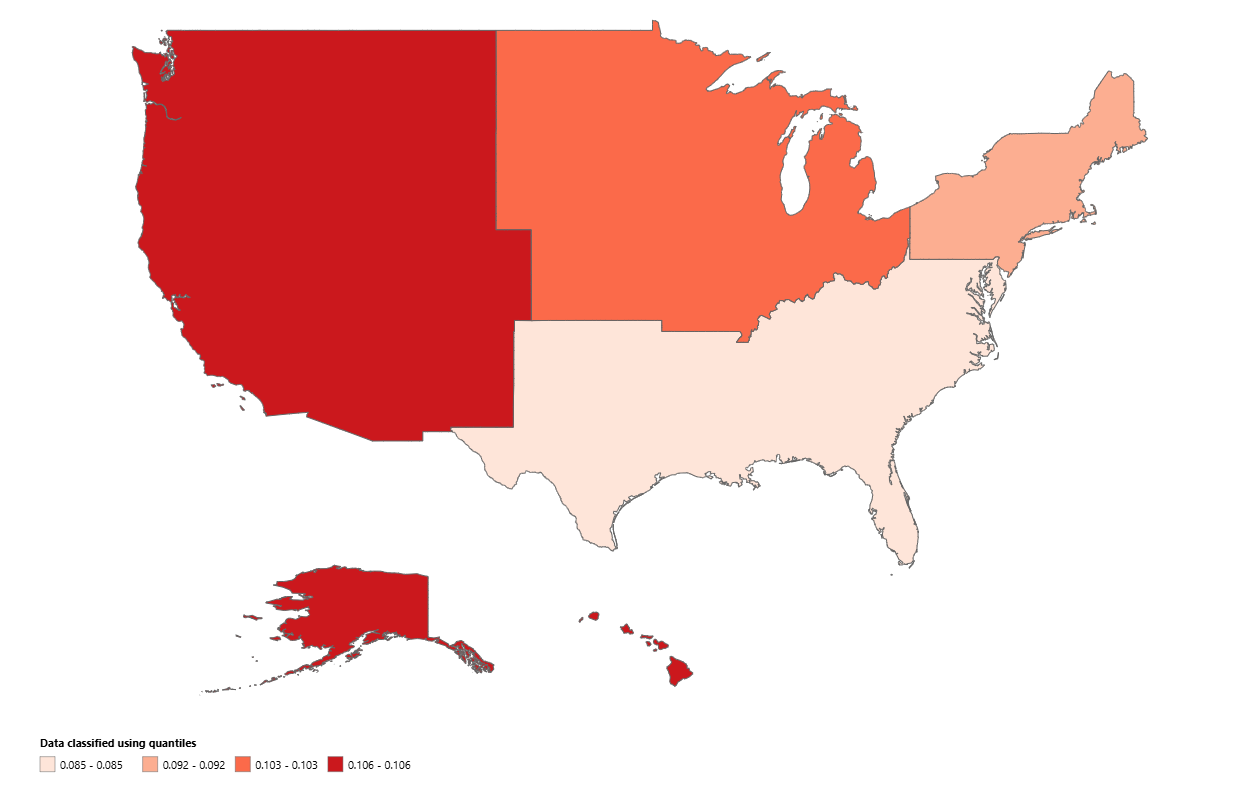


**Supplementary Figure 2:** Figure shows age-adjusted mortality rates per 100,000 individuals trends in atrial fibrillation and flutter-related mortality in patients with thyroid disorders stratified by census region in the United States from 1999 to 2020
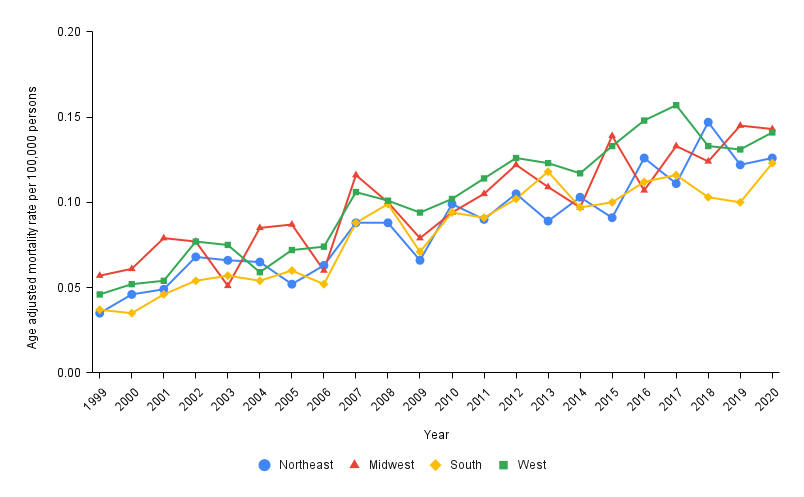


**Supplementary Figure 3:** Figure shows age-adjusted mortality rates per 100,000 individuals trends in atrial fibrillation and flutter-related mortality in patients with thyroid disorders stratified by urbanization in the United States from 1999 to 2020
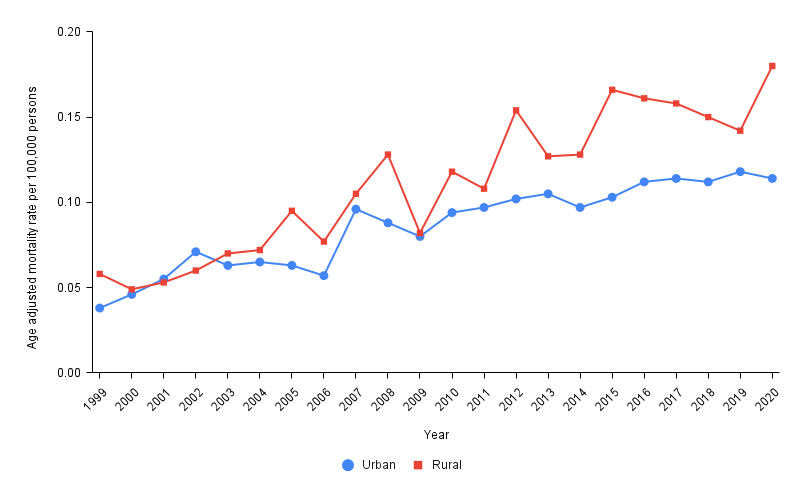


**Supplementary Figure 4:** Figure shows age-adjusted mortality rates per 100,000 individuals trends in atrial fibrillation and flutter-related mortality in patients with thyroid disorders stratified by ten-year age groups in the United States from 1999 to 2020
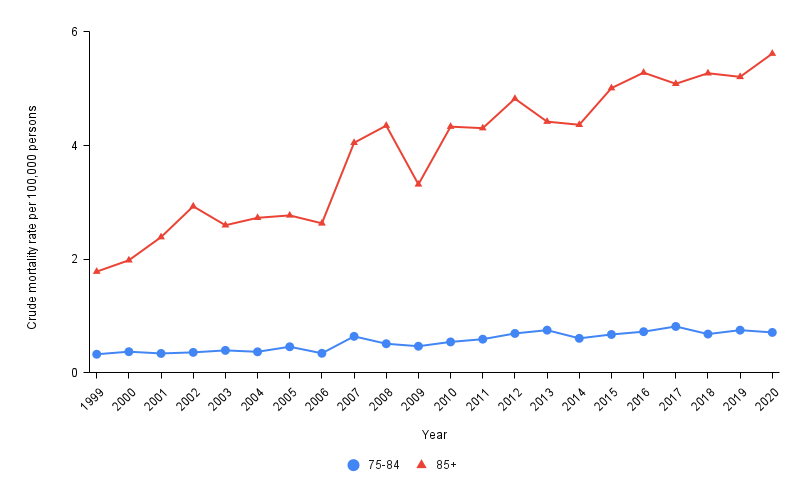

Supplement: Supplementary file 1 — Table S1. State‐wise age‐adjusted mortality rates per 100,000 individuals and 95% confidence intervals (95% CIs) in atrial fibrillation and flutter‐related mortality in patients with thyroid disorders in the United States from 1999 to 2020. Figure S1. Figure shows map of census region‐wise atrial fibrillation and flutter‐related mortality in patients with thyroid disorders in the United States from 1999 to 2020. Figure S2. Figure shows age‐adjusted mortality rates per 100,000 individuals trends in atrial fibrillation and flutter‐related mortality in patients with thyroid disorders stratified by census region in the United States from 1999 to 2020. Figure S3. Figure shows age‐adjusted mortality rates per 100,000 individuals trends in atrial fibrillation and flutter‐related mortality in patients with thyroid disorders stratified by urbanization in the United States from 1999 to 2020. Figure S4. Figure shows age‐adjusted mortality rates per 100,000 individuals trends in atrial fibrillation and flutter‐related mortality in patients with thyroid disorders stratified by 10‐year age groups in the United States from 1999 to 2020. [file JOA3-41-e70096-s001.docx]
